# Successful Aging in the North: Lessons Learned, Lessons Shared, and Advice From Those Aging in a Good Way in Alaska

**DOI:** 10.1093/geroni/igaa057.1369

**Published:** 2020-12-16

**Authors:** Jordan Lewis, Steffi Kim

**Affiliations:** 1 University of Minnesota Medical School, Duluth, Minnesota, United States; 2 University of Alaska Anchorage, Anchorage, Alaska, United States

## Abstract

This presentation presents and builds upon the Alaska Native Successful Aging model. Elders’ conceptions of successful aging differ from the Western model, how rural and urban environments influence successful aging, and the lessons Elders pass down to ensure a healthy lifestyle are not common in the literature. 42 Alaska Native Elders from the Norton-Sound sub region of Alaska, 21 Alaska Native Elders from the Aleutian Pribilof Islands, 26 Elders from the Bristol Bay region, and 12 Elders residing in Anchorage, AK to provide an urban context of successful aging. A community-based, exploratory, qualitative research methodology was used to allow for co-learning between research team and tribal communities. 101 qualitative interviews were conducted, and team-based thematic analysis was used to establish codes and main themes to expand the Alaska Native Successful Aging model. We learned that emotional well-being, community engagement, spirituality, family and purposeful engagement, and physical health were instrumental in promoting successful aging within Norton Sound and Bristol Bay. These findings expanded Lewis’s model to include gerotranscendence and generativity; Elders intentional relationships and stronger connections to traditional cultural and spiritual activities gave life deeper meaning and value. These findings argue the importance of including and emphasizing social components, historical perspective, and the importance of place, as well as generativity and gerotranscendence in program and policy development.

